# Meta-analysis of epigenome-wide association studies of carotid intima-media thickness

**DOI:** 10.1007/s10654-021-00759-z

**Published:** 2021-06-06

**Authors:** Eliana Portilla-Fernández, Shih-Jen Hwang, Rory Wilson, Jane Maddock, W. David Hill, Alexander Teumer, Pashupati P. Mishra, Jennifer A. Brody, Roby Joehanes, Symen Ligthart, Mohsen Ghanbari, Maryam Kavousi, Anton J. M. Roks, A. H. Jan Danser, Daniel Levy, Annette Peters, Sahar Ghasemi, Ulf Schminke, Marcus Dörr, Hans J. Grabe, Terho Lehtimäki, Mika Kähönen, Mikko A. Hurme, Traci M. Bartz, Nona Sotoodehnia, Joshua C. Bis, Joachim Thiery, Wolfgang Koenig, Ken K. Ong, Jordana T. Bell, Christine Meisinger, Joanna M. Wardlaw, John M. Starr, Jochen Seissler, Cornelia Then, Wolfgang Rathmann, M. Arfan Ikram, Bruce M. Psaty, Olli T. Raitakari, Henry Völzke, Ian J. Deary, Andrew Wong, Melanie Waldenberger, Christopher J. O’Donnell, Abbas Dehghan

**Affiliations:** 1 Department of Epidemiology, Erasmus University Medical Center, Rotterdam, The Netherlands; 2 Department of Internal Medicine, Division of Vascular Medicine and Pharmacology, Erasmus University Medical Center, Rotterdam, The Netherlands; 3 Population Sciences Branch, Division of Intramural Research, National Heart, Lung, and Blood Institute, National Institutes of Health, Bethesda, MD USA; 4 Framingham Heart Study, Framingham, MA USA; 5 Research Unit of Molecular Epidemiology, Institute of Epidemiology, Helmholtz Zentrum München, German Research Center for Environmental Health, Neuherberg, Germany; 6 MRC Unit for Lifelong Health and Ageing at UCL, Institute of Cardiovascular Science, University College London, London, UK; 7 Centre for Cognitive Ageing and Cognitive Epidemiology, University of Edinburgh, Edinburgh, UK; 8 Department of Psychology, University of Edinburgh, Edinburgh, UK; 9 Intitute for Community Medicine, University Medicine Greifswald, Greifswald, Germany; 10 DZHK (German Centre for Cardiovascular Research), Partner Site Griefswald, Greifswald, Germany; 11 Department of Clinical Chemistry, Fimlab Laboratories, and Finnish Cardiovascular Research Center - Tampere, Faculty of Medicine and Health Technology, Tampere University, Tampere, Finland; 12 Cardiovascular Health Research Unit, Department of Medicine, University of Washington, Seattle, WA USA; 13 Institute for Medical Information Processing, Biometry, and Epidemiology, Faculty of Medicine, Ludwig-Maximilians-Universität München, Munich, Germany; 14 German Center for Diabetes Research, Neuherberg, Germany; 15 Department of Neurology, University Medicine Greifswald, Greifswald, Germany; 16 Department of Internal Medicine B, University Medicine Greifswald, Greifswald, Germany; 17 Department of Psychiatry and Psychotherapy, University Medicine Greifswald, Greifswald, Germany; 18 Department of Clinical Physiology, Tampere University Hospital, and Finnish Cardiovascular Research Center - Tampere, Faculty of Medicine and Health Technology, Tampere University, Tampere, Finland; 19 Department of Microbiology and Immunology, Faculty of Medicine and Health Technology, Tampere University, Tampere, Finland; 20 Institute of Laboratory Medicine, Clinical Chemistry and Molecular Diagnostics, University Hospital, Leipzig, Leipzig, Germany; 21 DZHK (German Centre for Cardiovascular Research), Partner Site Munich Heart Alliance, Munich, Germany; 22 Deutsches Herzzentrum München, Technische Universität München, Munich, Germany; 23 Institute of Epidemiology and Medical Biometry, University of Ulm, Ulm, Germany; 24 MRC Epidemiology Unit and Department of Paediatrics, Wellcome Trust-MRC Institute of Metabolic Science, University of Cambridge School of Clinical Medicine, Cambridge, UK; 25 Department of Twin Research and Genetic Epidemiology, King’s College London, London, UK; 26 Independent Research Group, Clinical Epidemiology, Helmholtz Zentrum München - German Research Center for Environmental Health, Neuherberg, Germany; 27 Ludwig-Maximilians-Universität München, UNIKA-T, Augsburg, Germany; 28 Centre for Clinical Brain Sciences, University of Edinburgh, Edinburgh, UK; 29 Edinburgh Imaging, University of Edinburgh, Edinburgh, UK; 30 UK Dementia Research Institute, University of Edinburgh, Edinburgh, UK; 31 Diabetes Zentrum, Medizinische Klinik und Poliklinik IV – Campus Innenstadt, Klinikum Der Ludwig-Maximilians-Universität München, Munich, Germany; 32 Clinical Cooperation Group Diabetes, Ludwig-Maximilians-Universität München and Helmholtz Zentrum München, Munich, Germany; 33 Institute of Biometrics and Epidemiology, German Diabetes Center, Leibniz Institute at Heinrich Heine University Düsseldorf, Düsseldorf, Germany; 34 Department of Epidemiology, University of Washington, Seattle, WA USA; 35 Department of Health Services, University of Washington, Seattle, WA USA; 36 Kaiser Permanente Washington Health Research Institute, Seattle, WA USA; 37 Centre for Population Health Research, University of Turku and Turku University Hospital, Turku, Finland; 38 Research Centre of Applied and Preventive Cardiovascular Medicine, University of Turku, Turku, Finland; 39 Department of Clinical Physiology and Nuclear Medicine, Turku University Hospital, Turku, Finland; 40 Cardiology Section and Center for Population Genomics, VA Boston Healthcare System, Boston, MA USA; 41 Department of Medicine, Harvard Medical School, Boston, MA USA; 42 Department of Epidemiology and Biostatistics, School of Public Health, Faculty of Medicine, Imperial College London, Room 157, Norfolk Place, St Mary’s Campus, London, UK; 43 UK Dementia Research Institute at Imperial College London, London, UK; 44 MRC Centre for Environment and Health, School of Public Health, Imperial College London, London, UK

**Keywords:** Epigenome-wide association studies, Differentially methylated regions, DNA methylation, Common carotid intima-media thickness, Cardiovascular risk factors, Vascular outcomes, Mendelian randomization

## Abstract

Common carotid intima-media thickness (cIMT) is an index of subclinical atherosclerosis that is associated with ischemic stroke and coronary artery disease (CAD). We undertook a cross-sectional epigenome-wide association study (EWAS) of measures of cIMT in 6400 individuals. Mendelian randomization analysis was applied to investigate the potential causal role of DNA methylation in the link between atherosclerotic cardiovascular risk factors and cIMT or clinical cardiovascular disease. The CpG site cg05575921 was associated with cIMT (beta = −0.0264, *p* value = 3.5 × 10^–8^) in the discovery panel and was replicated in replication panel (beta = −0.07, *p* value = 0.005). This CpG is located at chr5:81649347 in the intron 3 of the aryl hydrocarbon receptor repressor gene (*AHRR*). Our results indicate that DNA methylation at cg05575921 might be in the pathway between smoking, cIMT and stroke. Moreover, in a region-based analysis, 34 differentially methylated regions (DMRs) were identified of which a DMR upstream of *ALOX12* showed the strongest association with cIMT (*p* value = 1.4 × 10^–13^)*.* In conclusion, our study suggests that DNA methylation may play a role in the link between cardiovascular risk factors, cIMT and clinical cardiovascular disease.

**Supplementary Information:**

The online version contains supplementary material available at 10.1007/s10654-021-00759-z.

## Introduction

Carotid intima-media thickness (cIMT) is defined as a progressive thickening of the arterial wall and is characterized by the presence of large arterial wall deposits. Hemodynamic changes leading to atherosclerotic plaques in both carotid and coronary arteries including lumen diameter, blood flow, shear stress, and tensile stress [1, 2]. Aetiological similarities in cardiovascular outcomes are also influenced by risk factors such as age, blood pressure, blood lipid and fasting plasma glucose which are independent predictors of carotid atherosclerosis [3, 4]. Evidence suggests that cIMT may be implicated as an intermediate factor in the causal pathway leading to cardiovascular disease [5] and can be used as a predictor of CAD and stroke. Ultrasound of the carotid artery is widely used as a non-invasive procedure to detect the presence of atherosclerotic plaques and as a marker of subclinical vascular disease [6]. A cIMT value above 75th percentile threshold for a person’s age, sex and race in asymptomatic individuals is associated with risk of myocardial infarction, stroke and death from CAD is significantly increased as compared to the average of the population [7]. The addition of cIMT to the Framingham Risk Score has been shown to improve the 10-year risk prediction of myocardial infarction or stroke independent of age, sex and cardiovascular risk factors [8]. Therefore, cIMT could add considerable utility to the study of the onset and progression of atherosclerosis [6].

Epigenetic modifications including covalent changes of DNA methylation and chromatin alterations, are known to determine genomic structure and to induce changes in the regulation of gene expression [9]. DNA methylation is considered as the most stable epigenetic mark and the most investigated in explaining gene expression patterns and cell differentiation. DNA methylation varies with age, sex and environmental factors including diet and smoking [10]. In recent years, there has been a growing interest in identifying whether DNA methylation variations contribute to the onset and progression of complex human diseases; accumulating evidence suggests that this is the case at least for some traits and disorders [11–15].

Technological advances and the implementation of epigenome-wide association study (EWAS), have facilitated the systematic assessment of DNA methylation signatures, leading to the identification of novel mechanisms related to human diseases [16–18]. Epigenomic profile characterized by EWAS has been assessed mainly in leukocytes since this is the most accessible tissue in epidemiologic studies. Although the sampling of the cell type mediating the disease allows more valid casual inference, it has been shown that the use of leukocytes, a more accessible surrogate cell type, yields useful information [12]. Given the established role of inflammation in development of CVD, differences in leucocyte DNA methylation patterns in healthy persons vs. those at risk could either reflect the cumulative effects of CVD risk factors or indicate the changes the leucocytes undergo in the course of developing CVD. The latter might be mimicking or reflect similar processes in vascular cells.

We assessed the association between DNA methylation markers and cIMT among over 6,000 European ancestry participants using data from eight cohorts participating in the Cohorts for Heart and Aging Research in Genomic Epidemiology (CHARGE) consortium. The analysis of epigenetic markers in relation to cIMT could provide insight into mechanisms related to arterial thickness and atherosclerotic disease. In addition, we analyzed and characterized differentially methylated regions (DMR) of the genome at which adjacent CpG sites show differential methylation levels across multiple samples. Information from multiple nearby methylation sites may aid biological inference as well as increase the power to detect associations with human traits [19].

## Methods

### Study population

**Fig. 1:**
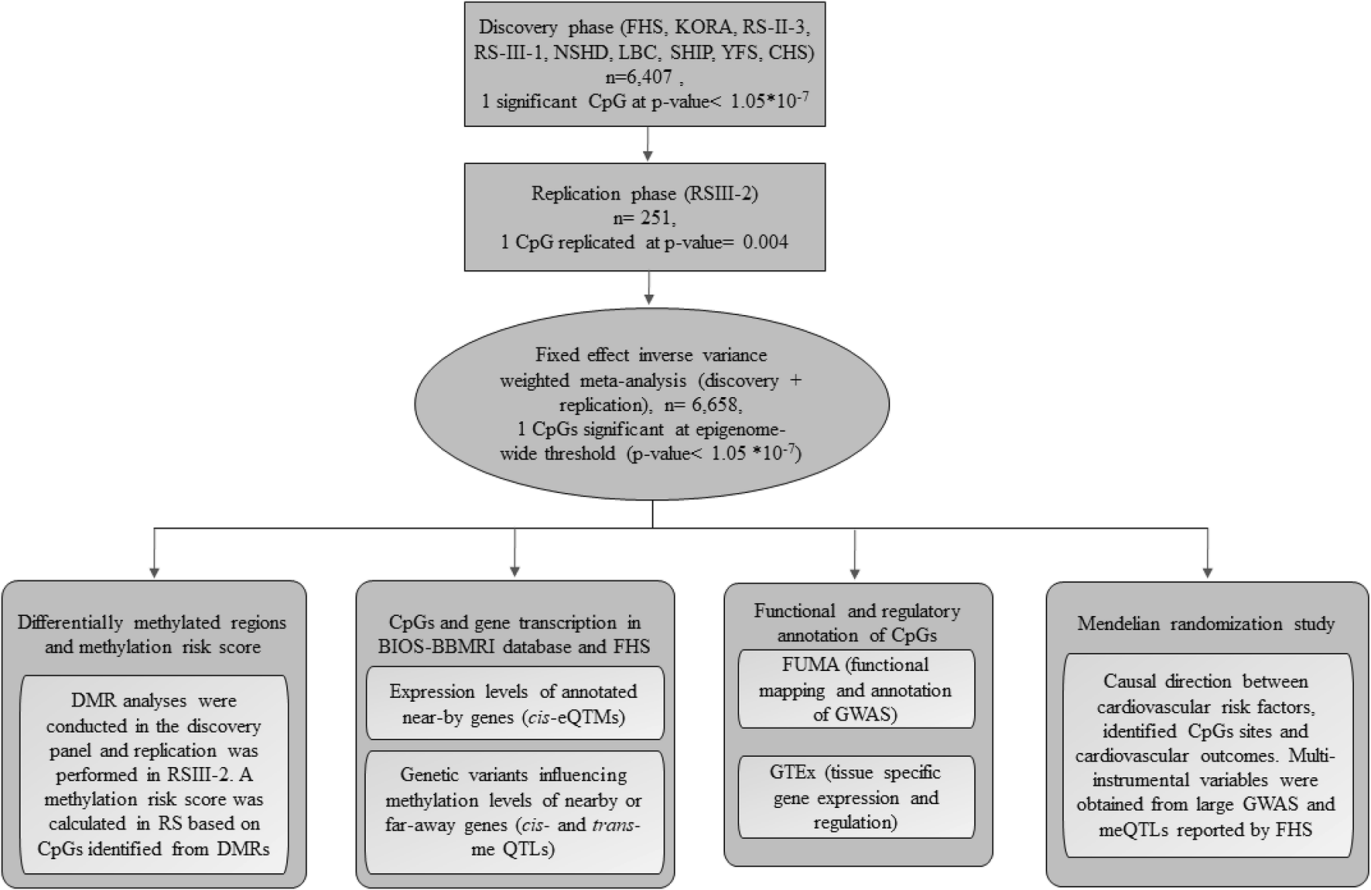
An overview of our study including EWAS meta-analysis to identify DNA methylation sites associated with carotid intima-media thickness, and post-EWAS in silico analyses. *FHS* Framingham Heart Study, *KORA* Cooperative Health Research in the Augsburg Region Study, *RS* Rotterdam study, *NSHD* MRC National Survey of Health and Development, *LBC* The Lothian Birth Cohorts, *SHIP* Study of Health in Pomerania, *YFS* Young Finns Study, *CHS* Cardiovascular Health Study

Figure 1 depicts an overview of the study flow. This study was conducted using data from eight cohorts within the Cohorts for Heart and Aging Research in Genomic Epidemiology (CHARGE) consortium, an international collaborative effort to facilitate collaborative efforts in omics era, providing opportunities for meta-analysis and replication among multiple studies [20]. The discovery panel comprised of 6407 subjects from Framingham Heart Study (FHS) (n = 1977, mean age ± SD = 66.2 ± 8.9), Cooperative Health Research in the Region Augsburg (KORA) (n = 1511, mean age ± SD = 60.7 ± 8.9), the Rotterdam Study (RS) (first visit of the third sub-cohort RSIII-1 (n = 731, mean age ± SD = 59.8 ± 8.2) and third visit of the second sub-cohort, RSII-3 (n = 468, mean age ± SD = 71 ± 3.2), MRC National Survey of Health and Development (NSHD) (n = 600, mean age ± SD = 53.4 ± 0.2), The Lothian Birth Cohorts (LBC) (n = 288, mean age ± SD = 72.1 ± 0.5), Study of Health in Pomerania (SHIP) (n = 246, mean age ± SD = 51.4 ± 13.8), Young Finns Study (YFS) (n = 191, mean age ± SD = 40.3 ± 3.3) and Cardiovascular Health Study (CHS) (n = 191, mean age ± SD = 76 ± 5) (Supplementary Table S1). For the replication panel, we used data from RSIII-2 (n = 251, mean age ± SD = 61.1 ± 4.3) (Supplementary Table S2). We performed a power calculation for the replication analysis using the GPower 3.1 tool [21]. Details of the participating studies are provided in Supplementary Appendix 1a. All participants provided written informed consent.

### Measurement of cIMT

All studies evaluated the carotid arteries of cohort participants using B-mode high-resolution ultrasound by trained operators. LBC, YFS, KORA, NSHD and SHIP cohorts obtained cIMT measurements from the far wall of the carotid artery; whereas CHS, FHS, and RS cohorts measured near and far walls. cIMT was calculated by averaging the maximum cIMT of the right and left common carotid artery in the mid portion of the visible segment of the arteries in the neck. A longitudinal image was used, optimizing the image frame to be perpendicular to the arterial wall. We used natural log transformation to deal with the skewness of the cIMT measurements. A description of the method employed in cIMT measurement by each study is provided in Supplementary Appendix 1a.

### DNA methylation measurements

Genome-wide DNA methylation profiling was conducted using Illumina Infinium BeadChips arrays. The cohorts FHS, KORA, RS, LBC, CHS and YFS used the HumanMethylation450 BeadChip®; which covers approximately 485,577 methylation sites. SHIP and NSHD employed Infinium MethylationEPIC BeadChip ® array, which covers approximately 850,000 CpG sites across the genome. Beta values, defined as the ratio of intensities between methylated and unmethylated CpG alleles were used to represent DNA methylation levels. Study-specific data pre-processing and quality control methods are outlined in Supplementary Table S3.

### Epigenome wide association analysis

All studies used linear mixed-effects models to identify associations between DNA methylation levels and cIMT. Two regression models were conducted. First, DNA methylation levels at each CpG site were regressed against natural-log transformed cIMT with adjustment for age, sex, white blood cell proportions (Houseman estimated proportions [22]), smoking history, microarray type and position (Model 1). Second, body mass index (BMI), HDL cholesterol/triglycerides ratio, systolic blood pressure, antihypertensive drug use, lipid lowering drug use and prevalent diabetes mellitus were added to the primary regression model (Model 2). Further details are provided in Supplementary Table S3. All statistical analyses were conducted using R [23].

### Meta-analysis of EWAS

Each cohort separately analyzed the EWAS of cIMT. Subsequently, EWAS summary statistics were uploaded to a central repository. Prior to their inclusion into the meta-analysis, all probes on sex chromosomes, non-CpG probes and cross-reactive probes were removed as suggested by Chen et al.[24]. The total number of probes included in the meta-analysis was 473,755. We performed a fixed effect inverse variance weighted meta-analysis using METAL [25]. A Bonferroni-corrected significance threshold (assuming 473,755 independent tests): 0.05/*n*_probes_max_ = 0.05/473,755 ≈ 1.05 × 10^−7^ was used to account for multiple comparisons and assumed as indicating epigenome-wide significance.

### Differentially methylated regions

The identification of DMR was conducted using Comb-p, a python library that combines and calculates the autocorrelation among adjacent *p* values found in genomic regions, in order to determine statistical significance at region level [26]. DMR analyses were conducted in the discovery panel and replication was performed in RSIII-2. Detailed description of DMR identification can be found in Supplementary Appendix 1b.

### Methylation risk score

A methylation risk score (MRS) was calculated for each participant of the Rotterdam Study based on DNA methylation patterns of CpGs identified from DMRs. We first developed the score in samples from RSIII-2 and RSII-3 and its performance was further tested in RSIII-1. The risk score was estimated as:

$$MRS = Bm_{1} *CpG_{1} + Bm_{2} *CpG_{2} + \ldots Bm_{k} *CpG_{k}$$

where *Bm* is the meta-analyzed effect of each CpG site on cIMT and k is the *kth* CpG. The MRS was calculated for each DMR (MRS.DMR) combined. We conducted a linear regression model using log-transformed cIMT as dependent variable and MRS for each genomic region (MRS.DMR) as independent variables adjusted for sex, age and smoking history. In the fully adjusted model, cell counts and batch effects were added to the fitted linear-mixed model using lme4 package [27]. Detailed information of the construction of MRS is outlined in Supplementary Appendix 1c.

### Integration of DNA methylation, genetic variation and gene expression

We examined the association of DNA methylation levels at the identified CpGs, with expression levels of their corresponding genes (*cis*-expression quantitative trait methylation (eQTM)) and genetic variants both in their vicinity, i.e. *cis*- and genome-wide i.e. *trans*-methylation quantitative trait loci (meQTL)using data from 4170 subjects from FHS cohort [28] and from five Dutch biobanks (BIOS-BBMRI database) (http://www.genenetwork.nl/biosqtlbrowser/). Significant threshold was set using Bonferroni-correction. For CpG-expression analysis, we corrected for 21,238 expression probes (α = 2.3 × 10^–6^ = 0.05/21,238). We also sought genetic variants influencing methylation levels of nearby or far-away genes.

### Functional and regulatory annotation of CpG sites

We used FUMA (Functional Mapping and Annotation of Genome-Wide Association Studies) [29] to functionally annotate cis-meQTLs. The SNP2GENE function was also used to test cis-meQTLs for association with other traits and diseases from the GWAS catalogue [30]. As epigenetic signatures are tissue dependent, and our analysis was limited in blood samples, we used expression quantitative trait loci (cis-eQTL) data from the Genotype-Tissue Expression Project (GTEx portal, Analysis Release V8) (http://www.gtexportal.org/home/) a platform with available expression data on potential target organs (heart tissue, kidney tissue, brain tissue, aortic endothelial cells and blood vessels) as well as blood cell types (CD4 + macrophages, monocytes).

#### Mendelian randomization analysis

We implemented a two-sample, two-step Mendelian Randomization (MR) study [31] (Supplementary Figure S1) to investigate for evidence of potentially causal relations between the identified CpG sites, cardiovascular risk factors and the risk of cardiovascular outcomes. First, we investigated whether the identified CpGs are causally affected by cardiovascular risk factors. We selected a panel of SNPs associated with each trait at a genome-wide level of significance (P < 5 × 10^–8^) and minor allele frequency > 0.01 as genetic instruments using published genome-wide association studies. Only studies including individuals of European ancestry were considered. We selected 167 genetic instruments for systolic blood pressure (SBP) [32, 33] (Supplementary Table S4), 170 SNPs associated with diastolic blood pressure (DBP) [32, 33] (Supplementary Table S5), 235 SNPs reported for pulse pressure (PP) [32, 33] (Supplementary Table S6), 123 variants associated with smoking index [34, 35] (Supplementary Table S7), 101 SNPs for low-density lipoprotein (LDL) cholesterol [36, 37] (Supplementary Table S8) and 40 SNPs associated with glucose levels [38] (Supplementary Table S9). We included 362 genetic variants previously found to be associated with BMI [39] (Supplementary Table S10). After adjustment for multiple testing using Bonferroni correction, the significance threshold was set at 0.01 taking into account the number of trait groups being tested (0.05/5 group traits including smoking, blood pressure, cholesterol, glucose and BMI). Second, we examined the effect of DNA methylation on cIMT, CAD and stroke. We chose instrumental variables for DNA methylation levels at c905575921 based on methylation quantitative trait loci (meQTL) obtained from FHS cohort (N = 4170) Details are described in Supplementary Methods and the list of instruments are presented in Supplementary Table S11 [28]. For step 2, we applied multiple testing correction based on three traits being evaluated and the *p* value threshold was set as 0.02. We used MR-PRESSO (Mendelian randomization pleiotropy residual sum and outlier) to identify horizontal pleiotropic outliers in multi-instrument summary-level MR testing (https://github.com/rondolab/MR-PRESSO) [40]. All MR methods for multiple genetic instruments were conducted using “MendelianRandomization”, a statistical package running under R (https://cran.rproject.org/web/packages/MendelianRandomization/index.html) [41]. Detailed description of MR methods is outlined in Supplementary Appendix 1.d.

## Results

Baseline characteristics of the nine discovery cohorts (n = 6157) and the replication cohort (n = 251) are presented in supplementary Tables S1 and S2. The sample sizes ranged from 191 to 1,977 individuals, all of European ancestry. Approximately half of participants were female, ranging from 47.6 to 62%. Mean ± SD age ranged from 40.3 ± 3.3 to 76.1 ± 5.1 years.

**Fig. 2:**
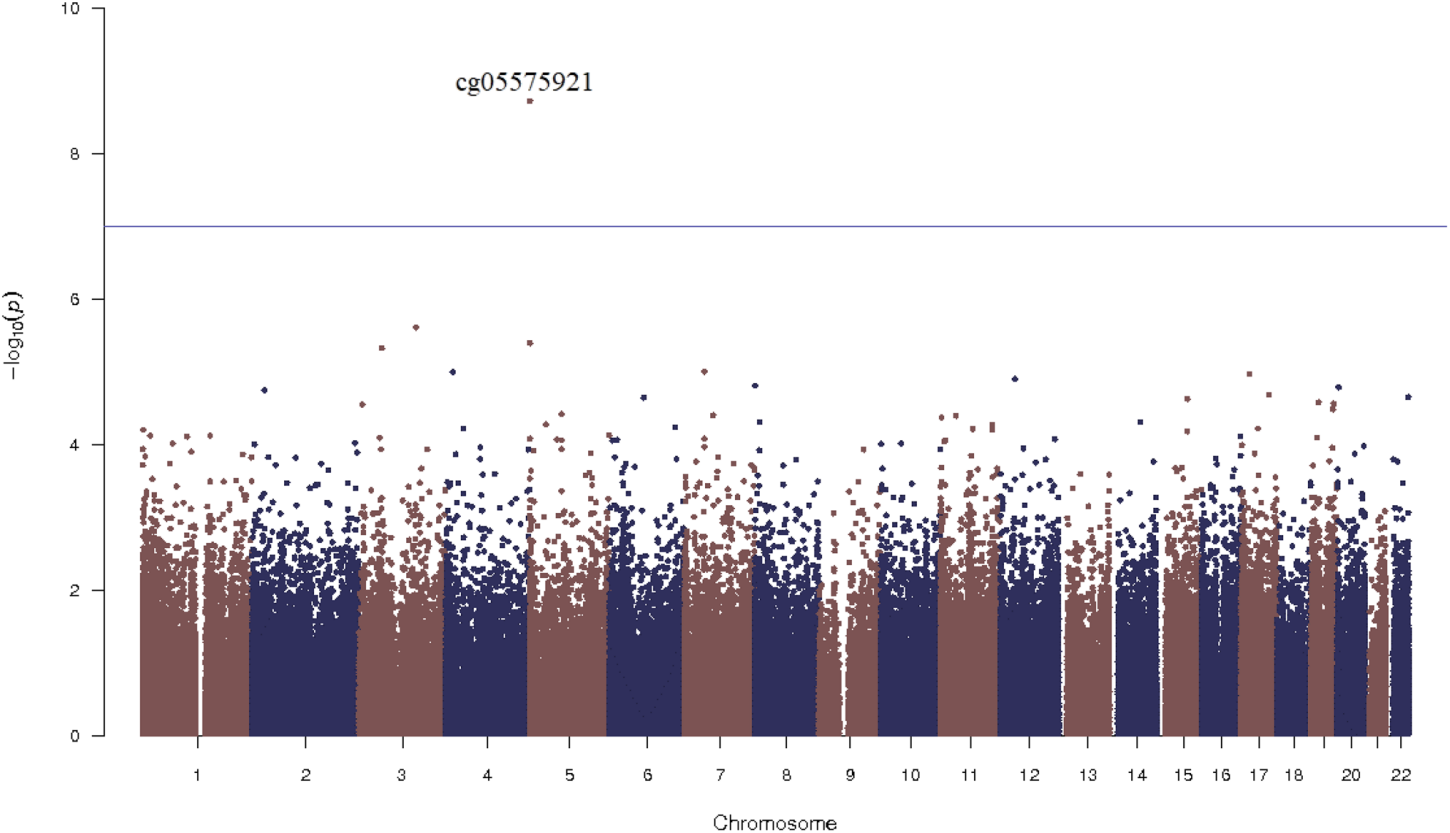
Manhattan plot Epigenome wide association between genome-wide DNA methylation and carotid intima media thickness

**Fig. 3:**
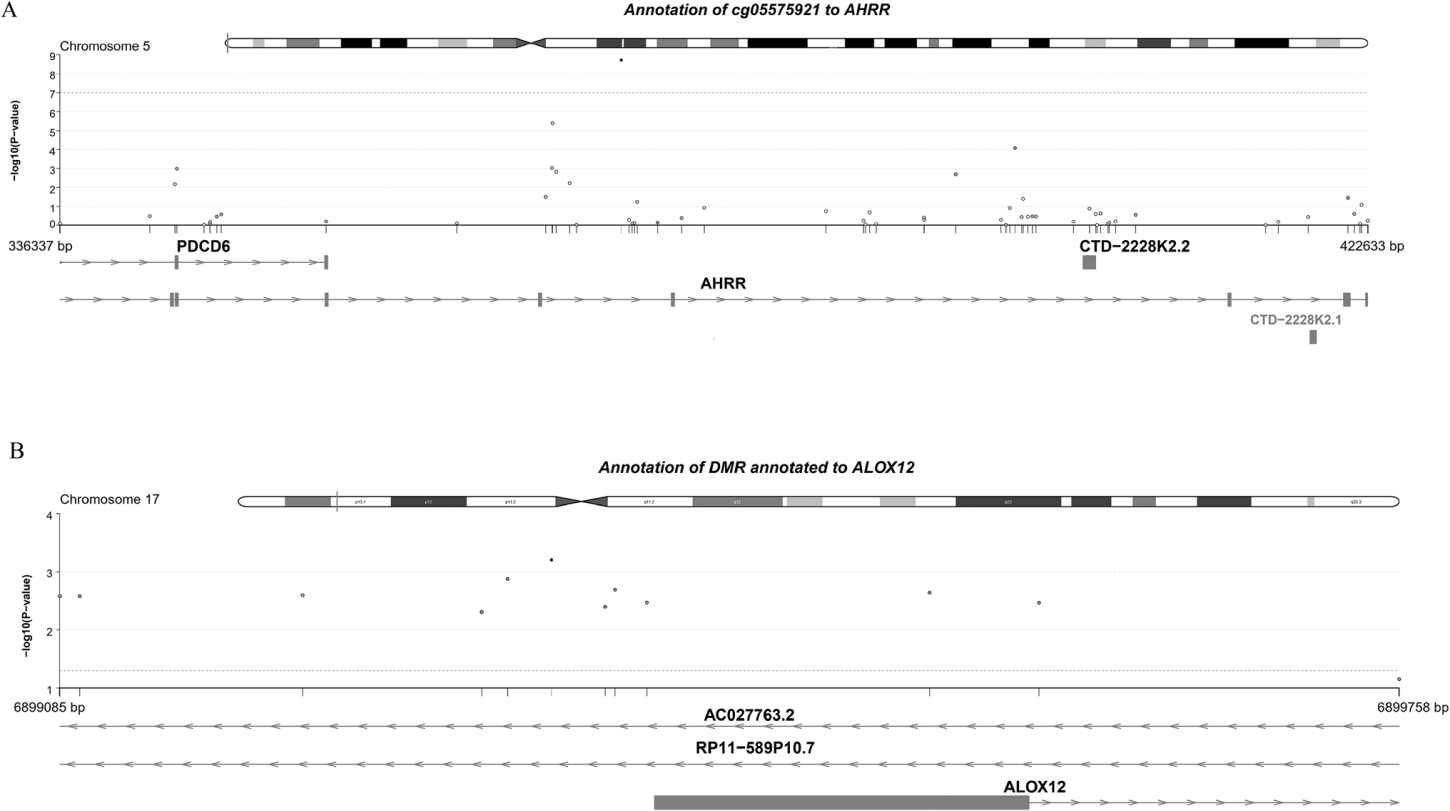
**a** Genomic location of cg05575921. **b** Genomic location of CpGs annotated to DMR *ALOX 12*

The quantile–quantile (QQ) plots were generated and corresponding lambda values computed for the overall meta-analysis of the discovery and replication panels combined, indicated no statistical inflation in Model 2. Figure 2 shows the Manhattan plot for the discovery meta-analysis of the model adjusted for age, sex, technical covariates, cell counts, smoking status, BMI, HDLC/TC ratio, SBP, antihypertensive and lipid lowering drugs and diabetes. There was one CpG associated with cIMT below the epigenome-wide-significance threshold (*p* value = 1.05 × 10^−7^). The CpG site cg05575921 showed the lowest *p* value = 2.2 × 10^–8^ (beta = −0.02, SE = 0.0048), and was replicated in RS-III2 with a *p* value = 0.004 (beta = −0.08) (Table 1). The cg05575921 CpG is located at chromosome 5 position: 81,649,347 in the intron 3 of the aryl hydrocarbon receptor repressor (*AHRR*) gene (Fig. 3a, [42]). Meta-analysis performed in smokers and non-smokers from Rotterdam Study revealed a stronger association between cg05575921 and cIMT in smokers, in comparison to non-smokers (smokers: beta = −0.07, *p* value = 8.1 × 10^–5^; non-smokers: beta = −0.01, *p* value = 0.2).Further adjustments for additional potential confounders including BMI, HDL cholesterol/triglycerides ratio, systolic blood pressure, antihypertensive and lipid lowering drug use, prevalent and diabetes mellitus did not substantially change the effect estimates and *p* values in both the discovery panel (*p* value = 2.2 × 10^–8^, beta = −0.02) and replication cohort (*p* value = 0.004, beta = −0.08) (Table 1). Furthermore, sensitivity analysis including hypertensive disease in the fully adjusted model showed no different result in comparison with Model 2.

**Table 1:** CpG sites significantly associated with CIMT in discovery panel and replication study

CpG site	Position	Gene	Discovery Panel		Replication study		Combined analysis	
			Beta	*p* value	Beta	*p* value	Beta	*p* value
cg05575921*	Chr5:373378	AHRR	− 0.02	3.5 × 10^−^^8^	− 0.07	0.005	− 0.03	3.17 × 10^−^^9^
cg05575921**	Chr5:373378	AHRR	− 0.02	2.2 × 10^−^^8^	− 0.08	0.004	− 0.03	1.9 × 10^−^^9^

*First model: BETA ~ Ln(cIMT) + age + sex + tech cov + cell counts + smoking status (+ study specific)

**Second model: BETA ~ Ln(cIMT) + age + sex + tech cov + cell counts + smoking status (+ study specific) + BMI + HDLC/TC ratio + SBP + antihypertensive + lipid lowering + pDM.

In the discovery panel, we identified 34 DMRs, composed of 247 CpGs, associated with cIMT. The strongest association (*p* value = 1.4 × 10^–13^) was observed in a cluster of 12 CpG sites annotated to the promoter region of *ALOX12* (in the upstream genomic region of chromosome 17 position: 6,899,085 to 6,899,759) (Supplementary Table S12, Fig. 3b). Most of the CpG sites clustered in these DMRs were annotated to the promoter region or transcription site of a close by genes. However, none of these DMRs were replicated in RSIII-2. To assess the variance of cIMT explained by these DMRS, we calculated a methylation risk score using 247 CpG sites. Fully adjusted linear regression models showed that MRS, which was developed in RSIII-2 and RSII-3, explained a small proportion of the cIMT variance (4.4%, *p* value = 2.2 × 10^–16^) observed in RSIII-1.

We further investigated the role of the methylation status in cg05575921 on gene expression levels measured in subjects from FHS. We found 285 gene expression markers associated with cg05575921 in *cis* and *trans* eQTM (Supplementary Table S13). The strongest association was found for the transcripts of the *LRRN3* (Leucine Rich Repeat Neuronal 3) gene. Hypermethylation status of cg05575921 was negatively correlated *LRRN3* (*p* value = 1.9 × 10^–100^). In the look-up for cis-eQTM in the BIOS-BBMRI dataset, cg05575921 was associated with expression levels of *EXOC3* (exocyst complex component 3) (*p* value = 1.2 × 10^–6^). These gene expression markers were not associated with cIMT, as measured in RSIII-1. Moreover, cg05575921 was associated with 116 genetic variants in the neighbouring genes including *AHRR, CCDC127, LRRC14B, PDCD6, PLEKHG4B* and *SDHA* (*cis*-meQTLs) (Supplementary Table S14). A heatmap depicting average expression of these 6 genes across 53 human tissues, available on the "Functional Mapping and Annotation of genetic associations with FUMA" webtool is provided in the Supplementary Figure S2. Based on the tissue specificity of differential expression using GTEXT, a total of 3,588 expression quantitative trait loci (eQTLs) in *AHRR* have been reported in several tissues, predominantly in brain (Supplementary Table S15, Supplementary Figure S3).

### Mendelian randomization

We applied a two-step Mendelian randomization analysis to investigate whether DNA methylation mediates the effect of traditional CVD risk factors on cardiovascular diseases. First, we studied the causal effect of cardiovascular risk factors on DNA methylation at cg05575921. Results from the conventional and sensitivity MR analyses are shown in Tables 2 and 3. MR estimates showed that smoking might have an effect on DNA methylation at cg05575921 (IVW·beta = −0.04, *p* value = 0.0001; Supplementary Figure S4). In contrast, MR estimates did not support an effect for LDL (IVW·beta = 0.002, *p* value = 0.4; Supplementary Figure S5), SBP (IVW·beta = −0.001, *p* value = 0.06; Supplementary Figure S6), DBP (IVW·beta = −0.001, *p* value = 0.3; Supplementary Figure S7), pulse pressure (IVW·beta = 0.0001, *p* value = 0.9; Supplementary Figure S8), glucose (IVW·beta = −0.01, *p* value = 0.3; Supplementary Figure S9) or BMI (IVW·beta = −0.008, *p* value = 0.07; Supplementary Figure S10). Second, we assessed whether there is any evidence for shared causal genetic variants between DNA methylation at cg05575921, cIMT, CAD and stroke. We identified 3 independent genetic instruments for this CpG site (*p* values < 5 × 10^–8^, R^2^ < 0.25). Only two out of the three genetic instruments were present in the meta-analysis of GWAS on CAD and no proxies have been reported for the absent SNP. We found that methylation at cg05575921 has a potential causal effect on cIMT (IVW·beta = 0.06, *p* value = 0.01; Supplementary Figure S11) and stroke (IVW·beta = 0.8, *p* value = 0.007; Supplementary Figure 12) but not on CAD (IVW·beta = 0.5, *p* value = 0.07).

**Table 2:** Mendelian randomization analysis on the effect of cardiovascular risk factors on cg05575921

Method	Estimate (95% CI)	*p* value	*p* value heterogeneity (IVW)
Smoking (no. cigarettes/day) → cg05575921			
IVW	− 0.04 (− 0.09 to 0.06)	0.0001	0.9
MR-Egger	− 0.06 (− 0.39 to −0.27)	0.08	–
MR-Egger (intercept)	0	0.5	–
Weighted median	− 0.04 (− 0.18 to 0.05)	0.08	–
LDL-cholesterol (mg/dl^−1^) → cg05575921			
IVW	0.002 (− 0.02 to 0.02)	0.4	0.9
MR-Egger	0.0001 (− 0.05 to 0.02)	0.9	–
MR-Egger (intercept)	0	0.5	–
Weighted median	0.003 (− 0.05 to 0.01)	0.4	–
Systolic blood pressure (mm Hg) → cg05575921			
IVW	− 0.001 (− 0.005 to 0.003)	0.06	0.4
MR-Egger	0.004 (− 0.01 to 0.01)	0.04	–
MR-Egger (intercept)	− 0.001	0.006	–
Weighted median	− 0.001 (− 0.008 to 0.004)	0.2	–
Diastolic blood pressure (mm Hg) → cg05575921			
IVW	− 0.001 (− 0.008 to 0.003)	0.3	0.8
MR-Egger	0.001 (0.026–0.07)	0.8	–
MR-Egger (intercept)	0	0.6	–
Weighted median	− 0.001 (− 0.01 to 0.007)	0.6	–
Pulse pressure (mm Hg) → cg05575921			
IVW	0.0001 (0.0001–0.002)	0.9	0.9
MR-Egger	0.0001 (0.0003–0.003)	0.8	–
MR-Egger (intercept)	0	0.8	–
Weighted median	− 0.001 (− 0.002 to 0.02)	0.5	–
Fasting glucose (mmol/l) → cg05575921			
IVW	− 0.01 (− 0.2 to 0.17)	0.3	0.6
MR-Egger	0.003 (0.005 to 0.008)	0.9	–
MR-Egger (intercept)	0	0.6	–
Weighted median	− 0.02 (− 0.3 to 0.2)	0.3	–
BMI (kg/m^2^) → cg05575921			
IVW	− 0.009 (− 0.002 to 0.034)	0.07	0.9
MR-Egger	0.001 (0.004− 0.008)	0.9	
MR-Egger (intercept)	0	0.4	
Weighted median	− 0.009 (− 0.002 to 0.04)	0.2	

*BMI* body mass index, *CAD* coronary artery disease, *cIMT* carotid intima media thickness, *IVW* inverse variance weighted, *LDL* low-density lipoprotein

**Table 3:** Mendelian randomization on the effect of cg05575921 on coronary artery disease (CAD), cIMT and stroke

Method	Estimate (95% CI)	*p* value	*p* value heterogeneity (IVW)
cg05575921 → CAD			
IVW	0.5 (− 0.03 to 0.9)	0.06	0.8
cg05575921 → cIMT (mm)			
IVW	0.06 (0.01–0.1)	0.01	0.2
MR-Egger	− 0.04 (− 0.2 to 0.09)	0.5	–
MR-Egger (intercept)	0.003	0.1	–
Weighted median	0.07 (0.008–0.13)	0.02	–
cg05575921 → Stroke			
IVW	0.8 (0.23–1.47)	0.007	0.8
MR-Egger	0.3 (− 1.3–1.9)	0.7	–
MR-Egger (intercept)	0.02	0.5	–
Weighted median	0.9 (0.17–1.58)	0.01	–

## Discussion

This study is the first epigenome wide association study on cIMT, an index of atherosclerosis. We report differential DNA methylation at one CpG site and 34 DMRs as associated with cIMT. The association found for the CpG cg05575921 was independent of potential confounders including BMI, lipid traits, blood pressure and smoking. The CpG sites found in DMRs combined in a methylation risk score, explained up to 4.4% of the variance observed in cIMT in European population. In addition, we found that DNA methylation at cg05575921 is implicated in the pathway between smoking, cIMT and stroke using Mendelian randomization analysis.

Our top hit is in the intronic region of the *AHRR* gene, located on chromosome 5 which is believed to possess several tumor suppressor genes [43, 44]. This gene encodes aryl-hydrocarbon receptor repressor, a protein that participates in the aryl hydrocarbon receptor (*AhR*) signaling cascade, which mediates the metabolism of xenobiotic particles like toxic cigarette smoke components [45, 46]. It functions as a feedback modulator by repressing AhR-dependent gene expression [47] and is also involved in regulation of cell growth and differentiation. While there is no clear mechanistic role of *AHRR* in atherogenesis, it is believed that numerous agonists of AhR signaling are contained in tobacco smoking and persistent activation of this signaling pathway may contribute to atherogenesis. Wu et al., showed that the treatment of macrophages with 2,3,7,8-tetrachlorodibenzo-p-dioxin (TCDD) [46] leads to AhR–dependent activation of inflammatory mediators and atherosclerotic plaque formation [48]. In addition, Vogel et al. demonstrated that TCDD promotes the differentiation of U937 macrophages to atherogenic foam cells, verified by lipid accumulation and extensive formation of blebs on the cell surface, which are characteristics of foam cells [49]. Meta-analysis performed in smokers and non-smokers from Rotterdam Study revealed a stronger association between cg05575921 and cIMT in smokers, in comparison to non-smokers. Based on our findings, smoking might explain the association described in this study We suggest the inclusion of more comprehensive covariates for smoking behavior (eg. Number of cigarettes per day, number of smoking years, passive smoking, etc.) in the modelling of DNA methylation-cIMT association.

We also observed an inverse association between DNA methylation of cg05575921 and levels of 285 mRNA expression probes, in which the majority are transcripts of inflammation genes. These findings suggest that increased methylation at *AHRR* gene decreases expression of relevant genes that are critical in the regulation of the inflammatory mechanisms taking place in the vascular wall. In our study, *LRRN3* gene showed the strongest association with methylation of cg05575921. The *LRRN3* has been shown to be differentially expressed between regions of plaque rich in smooth muscle cells and macrophages [50]. Furthermore, *LRRN3* has been incorporated in predictive models in whole blood to evaluate self-reported smoking status (current and recently quit smokers vs. former and never smokers) [51]. Based on our findings, the differentially expressed genes associated with cg05575921 could compromise downstream signals, resulting in the variability observed for cIMT in this population.

We also implemented bioinformatics tools to evaluate the presence of differentially methylated regions which are genomic regions with adjacent CpG islands that show differential methylation [19].The identification of DMRs is thought to provide a more comprehensive characterization of a genomic region based on the analysis of correlated CpGs [52]. In this study, the assessment of DMRs allowed us to identify associations of target genomic regions with cIMT. Among the regions identified, the strongest association was observed for *ALOX12*. Furthermore, significant associations were observed for 33 additional DMRs, including *AHRR,* located in genes involved in molecular mechanisms of cell signaling, vascular function and inflammation. *ALOX12* encodes a member of the lipoxygenase family of proteins. Lipoxygenases (LOXs) are dioxygenases that catalyze the formation of corresponding hydroperoxides from polyunsaturated fatty acids such as linoleic acid and arachidonic acid [53]. These bioactive lipid mediators are thought to exert potent actions on inflammatory reactions related to several cardiovascular diseases, such as atherosclerosis [54]. Polymorphisms in *ALOX12* have shown to be genetically associated with subclinical atherosclerosis and with biomarkers of disease in families with type 2 diabetes [55]. Mice models lacking *ALOX12* (P-12LO) exhibit a selective modulatory role for P-12LO in the ADP-induced pathway of platelet aggregation in mice, and increased mortality in an ADP-induced mouse model of thromboembolism [56]. Lipoxygenases, especially *ALOX12* may be considered as an interesting new genomic target for further investigations on traits related to vascular inflammation and impaired vascular function. Although these regions were not replicated on an independent sample, the genes identified have biologic relevance on the trait. The limited sample size of the replication cohort may have contributed to the lack of reproducibility of our findings.

The cross-sectional design of our study makes it difficult to determine whether cardiovascular risk factors are confounders or precursors in the reported methylation-cIMT associations. Causal inference in this setting can be addressed by Mendelian randomization methods, which may rule out reverse causation and confounding and provides further understanding on the direction of risk factor-outcome association. We conducted MR analysis addressing the effect of cardiovascular risk factors on DNA methylation and the effect of DNA methylation on cardiovascular outcomes. This enabled us to get a better understanding of the potential role of epigenetic markers in mediating the environmental impact on complex disease [31]. Results of our Mendelian randomization analysis suggest that smoking has a strong association on DNA methylation at cg05575921 which consecutively is associated with cIMT and stroke. Our MR findings strengthens the hypothesis regarding an effect of smoking on the methylation of the *AHRR* gene and its role in the mechanistic pathway between tobacco consumption and vascular outcomes. On the contrary, no MR association was observed for other cardiovascular risk factors included in this study. One explanation is that the genetic variants included in MR may explain a small proportion of the total variance in cardiovascular risk factors and DNA methylation status, and this could affect the statistical power to address any causal relations. Another explanation is that the risk factors do not interfere with the methylation of the CpG, but the CpG assists in increase of the risk, eg. by increasing the impact of smoking.

This study has several strengths and limitations. The major strength is the large sample size as it is thus far the largest EWAS meta-analyses exploring the association of DNA methylation with cIMT. All contributing cohorts had DNA methylation measured in whole blood, and adjustment for cell components allowed us to account for different epigenetic markers within cells present in the blood. Recent publications support that trait-specific differentially methylated sites identified in blood can show similar associations in the target tissue [33, 57, 58]. This suggests that DNA methylation measured in blood can be used as a proxy of methylation in vascular tissues, particularly given the established role of inflammation in development of atherosclerotic CVD. In addition, we implemented a wide variety of resources in the characterization of our findings, including gene expression assessment, identification of the effect of genetic variants on DNA methylation levels, identification of differentially methylated regions and the implementation of a comprehensive Mendelian randomization approach.

The findings of this study should be considered in light of some limitations. The meta-analysis results were obtained by combining DNA methylation results from European populations. The exclusion of 117 African-American (AA) individuals from CHS cohort was based on potential differences of DNA methylation patterns observed between individuals of AA and European ancestries. Indeed, the inclusion of data from non-European population led to different results in both single CpG meta-analysis and DMRs assessment. A sensitivity analysis to study the impact of ancestry in the results is a valuable approach, however our sample size was limited. Therefore, other studies are needed to assess the generalizability of our findings to other ancestries. Moreover, the causality results reported through Mendelian randomization methods should be addressed with caution. The variance explained by several SNPs associated with cg05575921 was rather small and the estimation of the strength of the instrumental variables yielded no suitable instruments for this CpG in Mendelian randomization analyses.

In conclusion, we identified one CpG located at *AHRR* to be associated with common carotid intima-media thickness, a subclinical marker of atherosclerosis. DNA methylation at *ALOX12* and other 33 DMRs also contribute to the phenotype. Furthermore, DNA methylation of *AHRR* gene might be implicated in the causal pathway between smoking, cIMT and stroke. Epigenetic changes may be useful as biomarkers of collective and accumulated exposures and diseases and when casual, as targets for modification through preventive and therapeutic interventions. The findings of our study could also have implications for prevention and treatement, however, we emphasize that our findings were modest in size and for the therapeutic use, the causality should be further studied including animal models, longitudinal studies of exposure-discordant monozygotic twins; and paying close attention to windows of vulnerability, environmental and nutritional assessment, and cell type-specific epigenetic patterns [59, 60].

## Supplementary Information

Below is the link to the electronic supplementary material.

Supplementary file1 (DOCX 736 kb)

Supplementary file2 (XLS 857 kb)

## Data Availability

The datasets generated during this study are available from the corresponding author upon reasonable request.
