# Supplementary material for: Meta-analysis of epigenome-wide association studies of carotid intima-media thickness
Source: Eur J Epidemiol. 2021 Jun 6;36(11):1143–55. doi: 10.1007/s10654-021-00759-z (PMC8629903; doi:10.1007/s10654-021-00759-z)
Supplement: Supplementary file 1 — Supplementary file1 (DOCX 736 kb) [file 10654_2021_759_MOESM1_ESM.docx]

**Supplementary Appendix**

**Meta-analysis of epigenome-wide association studies of carotid intima-media thickness**

**Table of Contents**

1. **Supplementary Methods:**
   1. Study-specific methods section.
   2. Assessment of differentially methylated regions
   3. Construction of methylation risk score
   4. Mendelian randomization analyses
2. **Supplementary Figures:**
   1. Figure S1. Two-step Mendelian randomization
   2. Figure S2. FUMA heatmap
   3. Figure S3. Expression quantitative trait loci of *AHRR* gene
   4. Figure S4. Mendelian Randomization plot smoking SNPs and cg05575921
   5. Figure S5. Mendelian Randomization plot LDL SNPs and cg05575921
   6. Figure S6. Mendelian Randomization plot systolic blood pressure SNPs and cg05575921
   7. Figure S7. Mendelian Randomization plot diastolic blood pressure SNPs and cg05575921
   8. Figure S8. Mendelian Randomization plot pulse pressure SNPs and cg05575921
   9. Figure S9. Mendelian Randomization plot glucose SNPs and cg05575921
   10. Figure S10. Mendelian Randomization plot body mass index SNPs and cg05575921
   11. Figure S11. Mendelian Randomization plot cg05575921 meQTLs and carotid intima-media thickness
   12. Figure S12. Mendelian Randomization plot cg05575921 meQTLs and stroke
3. **Funding and Acknowledgements**

**1. Supplementary Methods**

**a. Study-specific methods section**

**Cardiovascular Health Study**

*Cohort summary*

The Cardiovascular Health Study (CHS) is a population-based cohort study of risk factors for coronary heart disease and stroke in adults ≥65 years conducted across four field centers (1). The original predominantly European ancestry cohort of 5,201 persons was recruited in 1989-1990 from random samples of the Medicare eligibility lists; subsequently, an additional predominantly African-American cohort of 687 persons was enrolled for a total sample of 5,888. DNA methylation was measured on a randomly selected subset of 336 European ancestry and 329 African-American ancestry participants who participated in the 3rd annual follow-up visit (study year 5) and had DNA available from that visit. The European ancestry participants had no baseline history of coronary vascular disease (defined as coronary heart disease, congestive heart failure, peripheral vascular disease, valvular heart disease, stroke, or transient ischemic attack).

*Carotid intima media thickness measurement*

Carotid ultrasound was performed during the baseline clinic visit using Toshiba SSA-270A imaging units (Toshiba America Medical Systems, Tustin, CA) identically equipped with a phased-array imaging probe having a characteristic -3-dB cutoff point of 6.7 MHz and a pulsed Doppler frequency of 4.0 MHz. One longitudinal image of the distal 10 mm of the common carotid artery and 3 longitudinal images of the internal carotid artery were acquired. The maximal IMT of the common carotid artery was defined as the mean of the maximal IMT of the near and far walls on both the left and right sides. Focal plaques, when present, were included in the maximum IMT measurement.

**Cooperative Health Research in the Region Augsburg (KORA)**

*Cohort summary*

The KORA cohort ethical approval was granted by the ethics committee of the Bavarian Medical Association and was carried out in accordance with the principles of the Declaration of Helsinki. All research participants signed informed consent prior to taking part in any research activities. The KORA data protection procedures were approved by the responsible data protection officer of the Helmholtz Zentrum Munich (2).

*Carotid intima media thickness measurement*

Ultrasound measurements of participants' common carotid arteries were performed in KORA F4 by two certified investigators as described in (3). An average of the left and right cIMT was calculated and used as the outcome of interest in this analysis.

**The Lothian Birth Cohort 1936 (LBC1936)**

*Cohort summary*

LBC1936 (4) is a longitudinal cohort study composed of individuals, most of whom took part in the Scottish Mental Survey of 1947. It consists of 1091 (543 females). At recruitment most were living in and around Edinburgh when they were recruited to the LBC1936 between 2004 and 2007. Their mean age was 69.5 years (s.d. = 0.8). They were healthy, older individuals all of whom lived in the community. Full details of the collection and quality control (QC) steps undertaken on the LBC1936 methylation data have been reported previously (5).

*Carotid intima media thickness measurement*

Carotid ultrasound imaging was conducted using a Siemens Antares Premium Colour Doppler scanner (Siemens AG, Erlangen, Germany) and a 7.5 MHz variable frequency probe operated by experienced neurovascular ultrasonographers, all data were cross checked by a consultant neuroradiologist. Carotid IMT was recorded in the common carotid arteries (CCA) and the mean of three calliper measures from each artery for both the left and the right side. Full details have been published previously (6). The average of the left and the right was used as the outcome of interest in this study.

**Rotterdam Study**

*Cohort summary*

The Rotterdam Study (RS) is a prospective study, population-based cohort study ongoing since 1990 including population from the well-defined Ommoord district in the city of Rotterdam. Initially, the study included 7983 individuals 55 years aged or older. In 2000, 3011 additional participants who had become 55 years or moved into the study district were included to the cohort. In 2006, the RSIII cohort was established including 3932 subjects aged 45-54 years. As of 2008, the Rotterdam Study cohort comprises a total of 14,926 subjects aged 45 years All individuals comprised in this study were of European and African descent. The study has conducted extensive clinical examinations, repeated every 3–4 years, to investigate the causes and risk factors associated with cardiovascular diseases, cognitive-related disorders, respiratory diseases, liver diseases, diabetes mellitus, cancer among others (7).

*Carotid intima media thickness measurement*

Ultrasonography of both carotid arteries was performed with a 7.5 MHz linear-array transducer and a duplex scanner (ATL UltraMark IV; Advanced Technology Laboratories). Measurements of the common carotid artery (CCA) intima-media thickness involved a length of 10 mm distal of the bulb. cIMT was determined as the average of mean, near- and far-wall IMT, providing the average of left and right common carotid IMT.

**Study of health in Pomerania (SHIP-Trend)**

*Cohort summary*

The Study of Health in Pomerania (SHIP) is a longitudinal population-based cohort study in West Pomerania, a region in the northeast of Germany, assessing the prevalence and incidence of common population-relevant diseases and their risk factors. Baseline examinations for the SHIP-Trend cohort included in this project were carried out between 2008 and 2012, comprising 4,420 participants aged 20 to 81 years. Study design and sampling methods were previously described (8).

*Carotid intima media thickness measurement*

The far-wall intima-media thickness was assessed at the distal straight portion
of the right and left common carotid arteries (CCA) proximally from the
bifurcation using a semi-automated edge-tracking software, which provides the
maximal and the mean value of 250 measurements performed on a 10-mm segment of the CCA. The ‘mean of the maximum IMT’ was calculated by averaging the maximum IMT of both, the right and left CCA far wall.

**Framingham Heart Study (FHS)**

*Cohort summary*

The FHS sudy (Framingham Heart Study) is a longitudinal community-based family study.   FHS started in 1948 by recruitting residents of the town of Framingham, a suburban west of Boston Massachusetts, to conduct epidemiological studies for understanding the distribution and health burden of cardiovascular diseases and related risk factors.   The community-based study design succeed to characterize morbidity and mortality of cardiovascular diseases by data collection through clinical examinations, face to face interviews, laboratory tests, and routine follow-up through a study-specific clinic and routine survey contacts. In 1971, children and their spouses of the original participants were invited to participate the offspring study with every 4 years of follow-up examination. Offspring participants who attended the 8th follow-up examination with ultrasound examination for arterial wall thickness composed the study population of the cIMT project (9, 10).

*Carotid intima media thickness measurement*

Carotid intima media thickness was calculated through high resolution B-mode ultrasonohraphy readings.   For the current study we applied mean of the maximum thickness of the right and left common carotid atery (CCA) of the near and far walls.

**Young Finns Study (YFS)**

*Cohort summary*

The Cardiovascular Risk in Young Finns Study is an on-going multicentre follow-up study of atherosclerosis precursors of Finnish children and adolescents. The first cross-sectional survey was conducted in 1980. Total sample size was 4,320 children and adolescents aged 3, 6, 9, 12, 15 and 18 years. The subjects were randomly chosen from the national register. Total of 3,596 subjects (83.2 percent of those invited) participated in 1980. Follow-up studies have been conducted in 1983, 1986, 2001, 2007 and 2011 with the original cohort.

*Carotid intima media thickness measurement*

Ultrasound studies were performed using Sequoia 512 ultrasound mainframes (Acuson, CA, USA) with 13.0 MHz linear array transducers. Left carotid artery was scanned by ultrasound technicians following a standardised protocol. The image was focussed on the posterior (far) wall, and images were recorded from the angle showing the greatest distance between the lumen-intima interface and the media-adventitia interface. A scan including the beginning of the carotid bifurcation and the common carotid artery was recorded and stored in digital format on optical discs for subsequent off-line analysis. All scans were analysed by one reader blinded to subjects’ details. The best quality end-diastolic frame was selected. Several measurements of the common carotid far wall were taken approximately 10mm proximal to the bifurcation to derive maximal carotid IMT. To assess reproducibility of IMT measurements, we re-examined 60 subjects 3 months after the initial visit (2.5% random sample). The between-visit coefficient of variation of IMT measurements was 6.4%. To assess reproducibility of IMT image analysis, 113 scans were reanalysed by a second observer. The between observer coefficient of variation was 5.2%.

**MRC National Survey of Health and Development (NSHD)**

*Cohort summary*

The Medical Research Council (MRC) National Survey of Health and Development (or 1946 British birth cohort) originally consisted of 5362 babies born in one week in March 1946 to married parents in England, Scotland or Wales. This sample has been followed up twenty-four times since birth. At the 23rd follow-up (60-64 years), eligible study members (n=2856) were invited to complete a clinical assessment or home visitation by a nurse. A total of 2229 participants responded of whom 1690 attended a clinic where vascular measurements were conducted. Information about DNA methylation was collected when participants were 53 years (n=1376) and 60-64 years (n=694) (11-14).

*Carotid intima media thickness measurement*

cIMT was measured in the left and right common carotid artery using a high-resolution imaging scanner (Vivid I, GE Healthcare (12 MHz probe)) with a 12 MHz probe (15, 16). A combined average of left and right cIMT is used.

**b. Assessment of differentially methylated regions**

The identification of DMR was conducted using Comb-p, a python library that combines and calculates the autocorrelation among adjacent p-values found in genomic regions, in order to determine the level of significance at region level (17). Different algorithms provided by Comb-p tool were used to detect DMR. First, the autocorrelation function (ACF) was calculated based on fixed offsets of adjacent p-values at varying distance lags. We calculated CpG sites autocorrelation from 1 to 500 bases with a step size of 50 bases. Once the ACF was calculated, we adjusted each p-value according to adjacent p-values by using Stouffer–Liptak correction and using values from the ACF output (18). Enrichment regions or peaks on the p-values were identified by using the peak-finding algorithm and 0.05 to indicate a minimum p-value to start a region. Finally, we assigned a p-value for each identified region using the Stouffer–Liptak correction. A p-value smaller or equal to 0.05 was considered as statistically significant. We used a BED format input file sorted by chromosome and start position, which contained the meta-analyzed p-values for each marker.

**c. Construction of methylation risk score**

Methylation risk score was calculated based on the CpG sites located in differentially methylated regions (DMRs) identified from DMR analysis. CpGs for each region are highly correlated; therefore we gave priority to the most significant CpGs reported by Rotterdam Study RSIII-2 and RSII-3. The effect estimates from RSIII-2 and RSII-3 combined were used to build the methylation risk score. The score was further tested in RSIII-1. Linear regression was conducted using cIMT as the dependent variable and MRS independent variables. Models were adjusted for age, sex, blood cell counts, and technical covariates. With the use of linear regression models, we calculated the cIMT variance explained by the methylation risk score.

**d. Mendelian randomization analyses**

We implemented a two-step epigenetic Mendelian Randomization (MR) (19) to stablish the causal inferences about how DNA methylation at the identified CpG sites would alter the risk of vascular outcomes. In the first step, we investigated whether the identified CpGs are causally affected by cardiovascular risk factors including, systolic blood pressure, diastolic blood pressure, pulse pressure, smoking index, lipid fractions glucose and body mass index. Only continuous traits were considered, as MR analysis of a binary exposure using summary data in a two-sample setting can result in inflated causal estimates (20). We examined 167 genetic instruments for systolic blood pressure (SBP) (21, 22), 170 SNPs associated with diastolic blood pressure (DBP) (21, 22), 235 SNPs reported for pulse pressure (PP) (21, 22), 123 variants associated with smoking (23, 24), 101 SNPs for LDL (25, 26) and 40 SNPs associated with glucose levels (27). We included 362 genetic variants previously found to be associated with BMI, in a recent meta-analysis by the GIANT Consortium (28). Correlated SNPs were pruned (R^2^<0.01) and independent SNPs were used in the analyses. In the second step, we examined the effect of DNA methylation on CAD, cIMT and stroke. We chose instrumental variables for DNA methylation levels based on methylation quantitative trait loci (meQTL) obtained from FHS cohort (N=4170) (29). We applied the clumping procedure of PLINK (30) (LD pruning of SNPs with R^2^<0.25 within 250 kb), and obtained a set of three LD-independent *cis-*mQTL-SNPs which were subsequently used as instrumental variables. We tested the statistical significance of the association between the instrumental variable and each CpG site with the following formula:

$$F=\frac{R^{2}\left( n-1-k \right)}{\left( 1-R^{2} \right)\times k}$$

*R^2^* is the variance explained by the genetic instrument, n is the sample size used in the GWAs on DNA methylation and *k* is the number of variants. The R^2^ was estimated using the formula: (2× MAF (1-MAF) β^2^)/var (CpG), where β is the estimated effect of the genetic variant on DNA methylation and var(CpG) is the estimated variance (31). The strength of genetic instrumental variables was estimated based on a F statistics > 10, to avoid weak-instrument bias. The association of genetic instruments with CAD were obtained from the largest meta-analysis published by the UK-Biobank-Cardiogram (32) (<https://data.mendeley.com/datasets/2zdd47c94h/1>). The summary association statistics cIMT were obtained from collaboration between CHARGE consortium and the University College London-Edinburgh-Bristol (UCLEB) consortium (33) (https://www.ncbi.nlm.nih.gov/projects/gap/cgi-bin/study.cgi?study_id=phs000930.v6.p1; accession phs000930.v6.p1). The summary association statistics for stroke were obtained from the MEGASTROKE consortium (34). Mendelian randomization analyses were performed using inverse-variance weighted (IVW), weighted median estimates and MR-Egger method for multiple genetic instruments. We used MR-PRESSO (Mendelian randomization pleitropy residual sum and outlier) to identify horizontal pleiotropic outliers in multi-instrument summary-level MR testing (<https://github.com/rondolab/MR-PRESSO>) (35). All MR methods for multiple genetic instruments were conducted using “MendelianRandomization”, a statistical package running under R (36) (https://cran.r-project.org/web/packages/MendelianRandomization/index.html).

1. **Supplemental Figures:**

- 1. Figure S1. Two-step Mendelian randomization

Step 1

SNPs

CVD risk factors

CpG

cIMT, stroke, CAD

Step 2

CVD risk factors

CpG

CpG meQTLs

- 1.
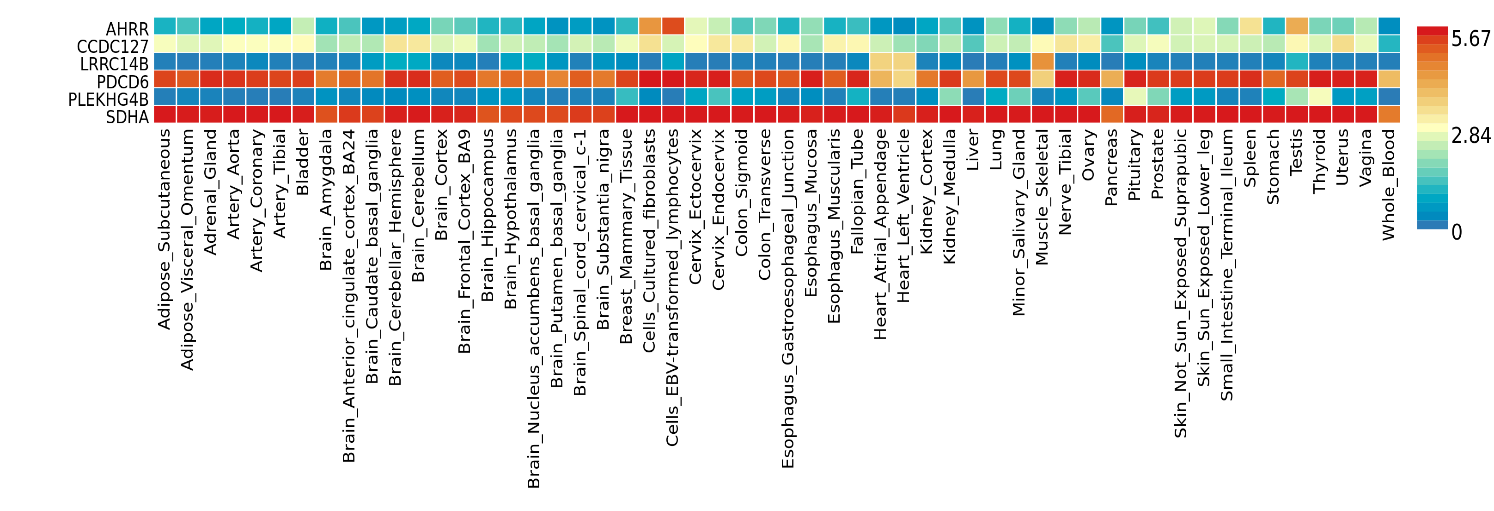
Figure S2.

Heatmap depicting an average expression of the genes, where the *cis*-meQTLs are annotated, in 53 specific tissue types provided by GTEx. Colors indicate the average expression value (log2 transformed Reads Per Kilobase per Million per tissue per gene, winsorization 50). Darker red color indicates higher expression of the gene, while darker blue represents lower expression level. This Figure is downloaded from the official GTEx through FUMA GWAS ([www.funa.ctglab.nl](http://www.funa.ctglab.nl)). GTEx=Genotype-Tissue Expression. FUMA GWAS= Functional Mapping and Annotation of Genome-wide Association Studies.

- 1.
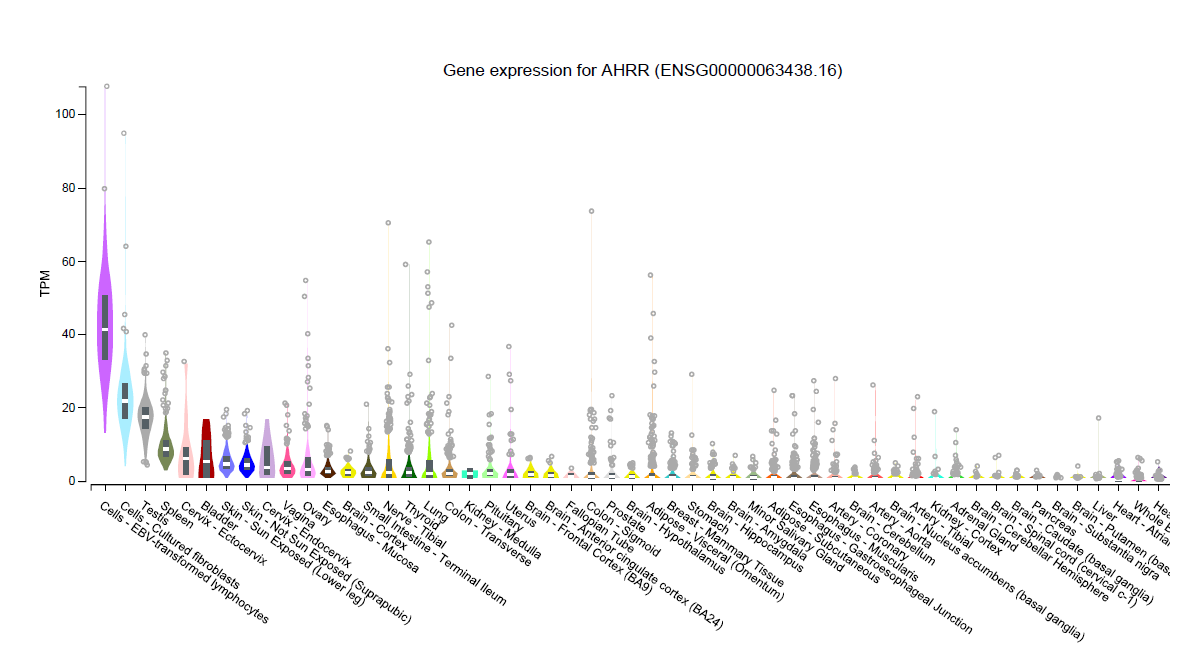
Figure S3.

Violin plot depicting expression quantitative trait loci associated to *AHRR.* Gene and transcript expression on the GTEx Portal are shown in Transcripts Per Million (TPM) and measured in 49 tissues. The values are linear scaled and sorted by the median. Each ‘violin’ represents the TPM estimates per tissue and the shape represents the density estimate of the expression levels. This Figure is downloaded from the official GTEx (https://www.gtexportal.org). GTEx=Genotype-Tissue Expression.

- 1. Figure S4. Mendelian Randomization plot smoking SNPs and cg05575921


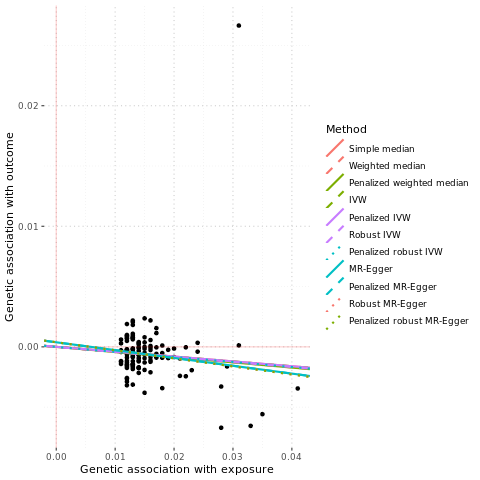


- 1. Figure S5. Mendelian Randomization plot LDL SNPs and cg05575921


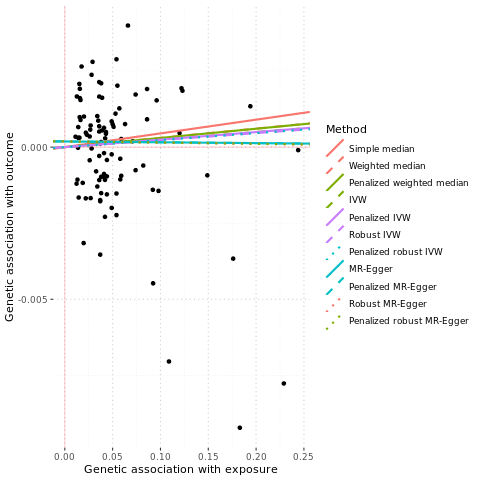


- 1. Figure S6. Mendelian Randomization plot systolic blood pressure SNPs and cg05575921


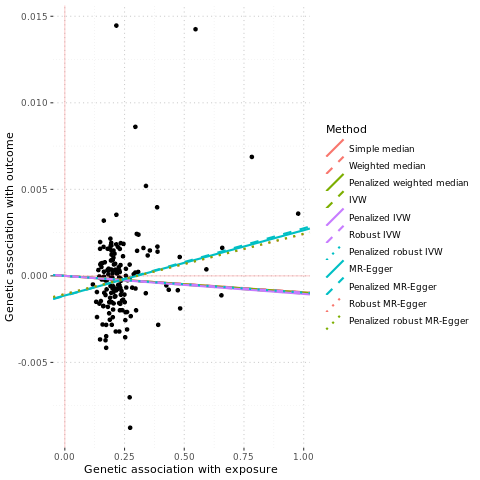


- 1. Figure S7. Mendelian Randomization plot diastolic blood pressure SNPs and cg05575921


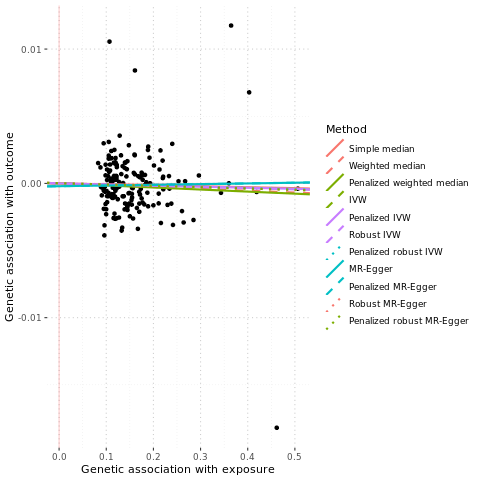


- 1. Figure S8. Mendelian Randomization plot pulse pressure SNPs and cg05575921


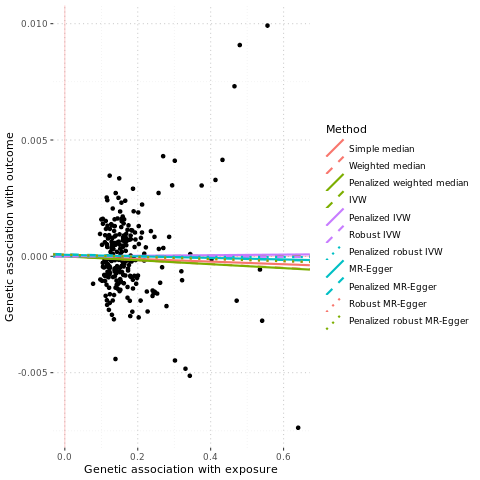


- 1. Figure S9. Mendelian Randomization plot glucose SNPs and cg05575921


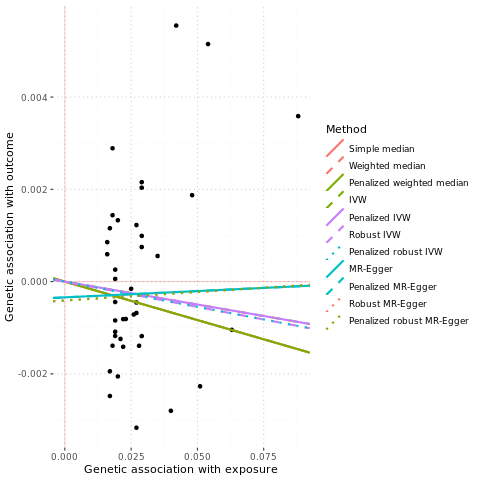


- 1. Figure S10. Mendelian Randomization plot body mass index SNPs and cg05575921


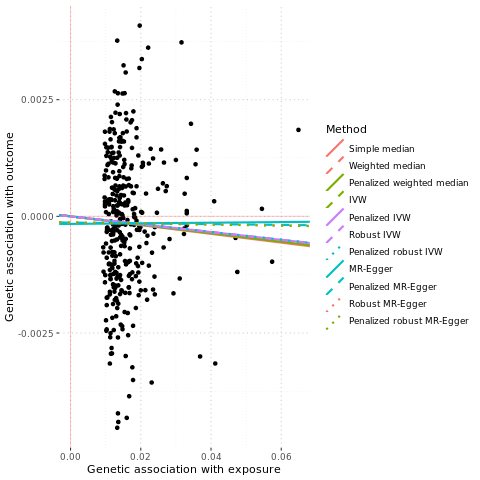


- 1. Figure S11. Mendelian Randomization plot cg05575921 meQTLs and carotid intima media thickness


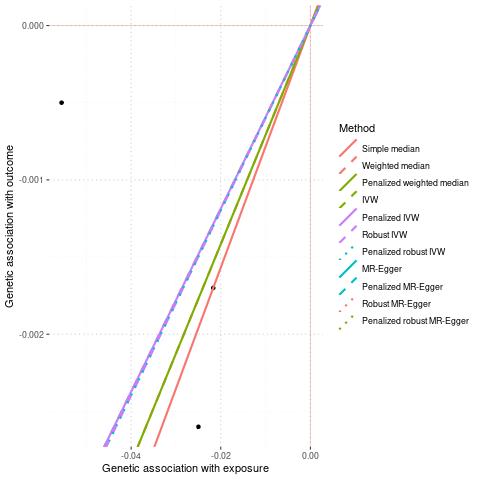


- 1. Figure S12. Mendelian Randomization plot cg05575921 meQTLs and stroke


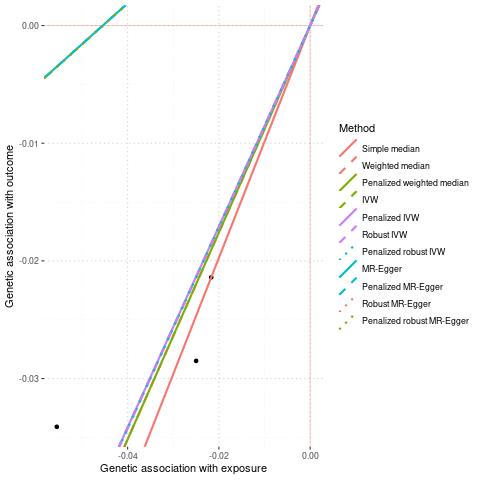


1. **Funding and Acknowledgements**

**Cardiovascular Health Study**

The CHS research was supported by NHLBI contracts 75N92021D00006, HHSN268201200036C, HHSN268200800007C, HHSN268201800001C, N01HC55222, N01HC85079, N01HC85080, N01HC85081, N01HC85082, N01HC85083, N01HC85086, N01HC85085, and N01HC45133; and NHLBI grants U01HL080295, U01HL130114, K08HL116640, R01HL087652, R01HL092111, R01HL103612, R01HL105756, R01HL103612, R01HL111089, R01HL116747 and R01HL120393 with additional contribution from the National Institute of Neurological Disorders and Stroke (NINDS). Additional support was provided through R01AG023629 from the National Institute on Aging (NIA), Merck Foundation / Society of Epidemiologic Research as well as Laughlin Family, Alpha Phi Foundation, and Locke Charitable Foundation. A full list of principal CHS investigators and institutions can be found at CHS-NHLBI.org. The provision of genotyping data was supported in part by the National Center for Advancing Translational Sciences, CTSI grant UL1TR000124, and the National Institute of Diabetes and Digestive and Kidney Disease Diabetes Research Center (DRC) grant DK063491 to the Southern California Diabetes Endocrinology Research Center. The content is solely the responsibility of the authors and does not necessarily represent the official views of the National Institutes of Health. Infrastructure for the CHARGE Consortium is supported in part by the National Heart, Lung, and Blood Institute grant R01HL105756.

**Cooperative Health Research in the Region Augsburg (KORA)**

The KORA study was initiated and financed by the Helmholtz Zentrum München – German Research Center for Environmental Health, which is funded by the German Federal Ministry of Education and Research (BMBF) and by the State of Bavaria. Furthermore, KORA research has been supported within the Munich Center of Health Sciences (MC-Health), Ludwig-Maximilians-Universität, as part of LMUinnovativ. This work was supported by a grant (WA 4081/1-1) from the German Research Foundation and by the German Federal Ministry of Education and Research (BMBF) within the framework of the EU Joint Programming Initiative ‘A Healthy Diet for a Healthy Life’ (DIMENSION grant number 01EA1902A).

**The Lothian Birth Cohort 1936 (LBC1936)**

The Lothian Birth Cohort research was supported by Age UK (Disconnected Mind grant), the Medical Research Council (Grant numbers MR/K026992/1, MR/R024065/1, MR/M013111/1, G1001245, 82800), the Biotechnology and Biological Sciences Research Council (BB/F019394/1), the University of Edinburgh, and the University of Queensland. We thank the LBC1936, the LBC1936 research team members, and the ultrasonographers. We thank Dr Riccardo Marioni and Professor Peter Visscher and his team for advice and help with DNA methylation data.

**Rotterdam Study**

This research was primarily supported by the Erasmus MC and Erasmus University Rotterdam; the Netherlands Organization for Scientific Research (NWO); the Netherlands Organization for Health Research and Development (ZonMw); the Research Institute for Diseases in the Elderly (RIDE); the Netherlands Genomics Initiative (NGI); the Ministry of Education, Culture and Science, the Ministry of Health, Welfare and Sports; the European Commission (DG XII); and the Municipality of Rotterdam. This work was also supported by Dutch Heart Foundation [grant number 2015T094 (to A.J.M.R)]. The contribution of the inhabitants, general practitioners, and pharmacists of the Ommord district to the Rotterdam Study is gratefully acknowledge.

**Study of health in Pomerania (SHIP-Trend)**

The Study of Health in Pomerania (SHIP) is part of the Community Medicine Research net (CMR) (http://www.medizin.uni-greifswald.de/icm) of the University Medicine Greifswald, which is supported by the German Federal State of Mecklenburg- West Pomerania. Genome-wide genotyping in SHIP-TREND has been supported by a joint grant from Siemens Healthineers, Erlangen, Germany and the Federal State of Mecklenburg-West Pomerania.

**Framingham Heart Study (FHS)**

Support for FHS was provided by the National Institutes of Health contract N01-HC-25195 (Boston University). We would also like to thank the FHS participants and families who participate in the FHS researchers. In addition we acknowledge the contributions of the research scientists and staffs of the Division of Intramural Research, National Heart, Lung, and Blood Institute (NHLBI), National Institutes of Health (NIH) and Boston University.

**Young Finns Study (YFS)**

The Young Finns Study has been financially supported by the Academy of Finland (grants 255381, 256474, 283115, 302382, 319060, 320297, 314389 (TERVA), 322098, 286284, 134309 (EYE), 126925, 121584, 124282, 129378 (SALVE), 117797 (GENDI), 273971 (TULOS) and 141071 (SKIDI); the Ministry of Education and Culture, Finland; the Social Insurance Institution of Finland; Competitive State Research Financing of the Expert Responsibility area of Kuopio, Tampere and Turku University Hospital grants; Juho Vainio Foundation; Paavo Nurmi Foundation; Finnish Foundation for Cardiovascular Research; Finnish Cultural Foundation; The Sigrid Juselius Foundation; Tampere Tuberculosis Foundation; Emil Aaltonen Foundation; Yrjö Jahnsson Foundation; Signe and Aune Gyllenberg Foundation; Päivikki and Sakari Sohlberg Foundation; Finnish Sport Institute Foundation; Diabetes Research Foundation of Finnish Diabetes Association; EU Horizon 2020 (grant 755320 for TAXINOMISIS); European Research Council (grant 742927 for MULTIEPIGEN project); grant 755320 for TAXINOMISIS; grant 848146 for TO-AITION;and Tampere University Hospital Supporting Foundation.

**MRC National Survey of Health and Development (NSHD)**

The UK Medical Research Council provides core funding for the MRC National Survey of Health and Development [MC_UU_00019/1]. We thank NSHD study members for their lifelong participation and past and present members of the study teams, including members of the MRC Epidemiology unit in Cambridge, and Kings College London who helped to collect and process the DNA methylation data. Data used in this publication are available to bona fide researchers upon request to the NSHD Data Sharing Committee via a standard application procedure. Further details can be found at   [http://www.nshd.mrc.ac.uk/data](https://eur01.safelinks.protection.outlook.com/?url=http%3A%2F%2Fwww.nshd.mrc.ac.uk%2Fdata&data=02%7C01%7Ce.portillafernandez%40erasmusmc.nl%7C03b156e8c0e746778d3a08d8110d7124%7C526638ba6af34b0fa532a1a511f4ac80%7C0%7C0%7C637278096714875381&sdata=p1IFBOqSBYkJc9AxOoJT8Ni0Vd4OKe2zGhArUgL%2BMAo%3D&reserved=0) doi:10.5522/NSHD/Q101,doi:10.5522/NSHD/Q102, doi: 10.5522/NSHD/S201.

References

1. Fried LP, Borhani NO, Enright P, Furberg CD, Gardin JM, Kronmal RA, et al. The cardiovascular health study: design and rationale. Annals of epidemiology. 1991;1(3):263-76.

2. Holle R, Happich M, Löwel H, Wichmann H. KORA--a research platform for population based health research. Gesundheitswesen (Bundesverband der Arzte des Offentlichen Gesundheitsdienstes (Germany)). 2005;67:S19-25.

3. Kowall B, Ebert N, Then C, Thiery J, Koenig W, Meisinger C, et al. Associations between blood glucose and carotid intima-media thickness disappear after adjustment for shared risk factors: the KORA F4 study. PLoS One. 2012;7(12):e52590.

4. Taylor AM, Pattie A, Deary IJ. Cohort profile update: the Lothian Birth Cohorts of 1921 and 1936. International journal of epidemiology. 2018;47(4):1042-r.

5. Shah S, McRae AF, Marioni RE, Harris SE, Gibson J, Henders AK, et al. Genetic and environmental exposures constrain epigenetic drift over the human life course. Genome research. 2014;24(11):1725-33.

6. Wardlaw JM, Allerhand M, Eadie E, Thomas A, Corley J, Pattie A, et al. Carotid disease at age 73 and cognitive change from age 70 to 76 years: A longitudinal cohort study. Journal of Cerebral Blood Flow & Metabolism. 2017;37(8):3042-52.

7. Hofman A, Brusselle GG, Murad SD, van Duijn CM, Franco OH, Goedegebure A, et al. The Rotterdam Study: 2016 objectives and design update. European journal of epidemiology. 2015;30(8):661-708.

8. Volzke H, Alte D, Schmidt CO, Radke D, Lorbeer R, Friedrich N, et al. Cohort profile: the study of health in Pomerania. Int J Epidemiol. 2011;40(2):294-307.

9. Dawber TR, Kannel WB. The Framingham Study an epidemiological approach to coronary heart disease. Circulation. 1966;34(4):553-5.

10. Feinleib M, Kannel WB, Garrison RJ, McNamara PM, Castelli WP. The Framingham offspring study. Design and preliminary data. Preventive medicine. 1975;4(4):518-25.

11. Wadsworth M, Kuh D, Richards M, Hardy R. Cohort profile: the 1946 national birth cohort (MRC National Survey of Health and Development). International journal of epidemiology. 2005;35(1):49-54.

12. Kuh D, Pierce M, Adams J, Deanfield J, Ekelund U, Friberg P, et al. Cohort profile: updating the cohort profile for the MRC National Survey of Health and Development: a new clinic-based data collection for ageing research. International journal of epidemiology. 2011;40(1):e1-e9.

13. Kuh D, Wong A, Shah I, Moore A, Popham M, Curran P, et al. The MRC National Survey of Health and Development reaches age 70: maintaining participation at older ages in a birth cohort study. European journal of epidemiology. 2016;31(11):1135-47.

14. Stafford M, Black S, Shah I, Hardy R, Pierce M, Richards M, et al. Using a birth cohort to study ageing: representativeness and response rates in the National Survey of Health and Development. European journal of ageing. 2013;10(2):145-57.

15. Masi S, D'Aiuto F, Martin-Ruiz C, Kahn T, Wong A, Ghosh AK, et al. Rate of telomere shortening and cardiovascular damage: a longitudinal study in the 1946 British Birth Cohort. European heart journal. 2014;35(46):3296-303.

16. Charakida M, Khan T, Johnson W, Finer N, Woodside J, Whincup PH, et al. Lifelong patterns of BMI and cardiovascular phenotype in individuals aged 60–64 years in the 1946 British birth cohort study: an epidemiological study. The lancet Diabetes & endocrinology. 2014;2(8):648-54.

17. Pedersen BS, Schwartz DA, Yang IV, Kechris KJ. Comb-p: software for combining, analyzing, grouping and correcting spatially correlated P-values. Bioinformatics. 2012;28(22):2986-8.

18. Kechris KJ, Biehs B, Kornberg TB. Generalizing moving averages for tiling arrays using combined p-value statistics. Statistical applications in genetics and molecular biology. 2010;9(1).

19. Relton CL, Davey Smith G. Two-step epigenetic Mendelian randomization: a strategy for establishing the causal role of epigenetic processes in pathways to disease. International journal of epidemiology. 2012;41(1):161-76.

20. Disney-Hogg L, Cornish AJ, Sud A, Law PJ, Kinnersley B, Jacobs DI, et al. Impact of atopy on risk of glioma: a Mendelian randomisation study. BMC Med. 2018;16(1):42.

21. Evangelou E, Warren HR, Mosen-Ansorena D, Mifsud B, Pazoki R, Gao H, et al. Genetic analysis of over 1 million people identifies 535 new loci associated with blood pressure traits. Nature genetics. 2018;50(10):1412.

22. Warren HR, Evangelou E, Cabrera CP, Gao H, Ren M, Mifsud B, et al. Genome-wide association analysis identifies novel blood pressure loci and offers biological insights into cardiovascular risk. Nature genetics. 2017;49(3):403.

23. Furberg H, Kim Y, Dackor J, Boerwinkle E, Franceschini N, Ardissino D, et al. Genome-wide meta-analyses identify multiple loci associated with smoking behavior. Nature genetics. 2010;42(5):441.

24. Wootton RE, Richmond RC, Stuijfzand BG, Lawn RB, Sallis HM, Taylor GMJ, et al. Causal effects of lifetime smoking on risk for depression and schizophrenia: Evidence from a Mendelian randomisation study. bioRxiv. 2018:381301.

25. Willer CJ, Schmidt EM, Sengupta S, Peloso GM, Gustafsson S, Kanoni S, et al. Discovery and refinement of loci associated with lipid levels. Nat Genet. 2013;45(11):1274-83.

26. Klarin D, Damrauer SM, Cho K, Sun YV, Teslovich TM, Honerlaw J, et al. Genetics of blood lipids among~ 300,000 multi-ethnic participants of the Million Veteran Program. Nature genetics. 2018;50(11):1514.

27. Dupuis J, Langenberg C, Prokopenko I, Saxena R, Soranzo N, Jackson AU, et al. New genetic loci implicated in fasting glucose homeostasis and their impact on type 2 diabetes risk. Nat Genet. 2010;42(2):105-16.

28. Yengo L, Sidorenko J, Kemper KE, Zheng Z, Wood AR, Weedon MN, et al. Meta-analysis of genome-wide association studies for height and body mass index in∼ 700000 individuals of European ancestry. Human molecular genetics. 2018;27(20):3641-9.

29. Huan T, Joehanes R, Song C, Peng F, Guo Y, Mendelson M, et al. Genome-wide identification of DNA methylation QTLs in whole blood highlights pathways for cardiovascular disease. Nature communications. 2019;10(1):1-14.

30. Chang CC, Chow CC, Tellier LCAM, Vattikuti S, Purcell SM, Lee JJ. Second-generation PLINK: rising to the challenge of larger and richer datasets. Gigascience. 2015;4(1):s13742-015-0047-8.

31. Ligthart S, Vaez A, Võsa U, Stathopoulou MG, De Vries PS, Prins BP, et al. Genome analyses of> 200,000 individuals identify 58 loci for chronic inflammation and highlight pathways that link inflammation and complex disorders. The American Journal of Human Genetics. 2018;103(5):691-706.

32. van der Harst P, Verweij N. The Identification of 64 Novel Genetic Loci Provides an Expanded View on the Genetic Architecture of Coronary Artery Disease. Circulation research. 2017:CIRCRESAHA. 117.312086.

33. Franceschini N, Giambartolomei C, de Vries PS, Finan C, Bis JC, Huntley RP, et al. GWAS and colocalization analyses implicate carotid intima-media thickness and carotid plaque loci in cardiovascular outcomes. Nat Commun. 2018;9(1):5141.

34. Malik R, Chauhan G, Traylor M, Sargurupremraj M, Okada Y, Mishra A, et al. Multiancestry genome-wide association study of 520,000 subjects identifies 32 loci associated with stroke and stroke subtypes. Nature genetics. 2018;50(4):524-37.

35. Verbanck M, Chen C-Y, Neale B, Do R. Detection of widespread horizontal pleiotropy in causal relationships inferred from Mendelian randomization between complex traits and diseases. Nature genetics. 2018;50(5):693.

36. Yavorska OO, Burgess S. MendelianRandomization: an R package for performing Mendelian randomization analyses using summarized data. International journal of epidemiology. 2017;46(6):1734-9.
